# Rinsing solutions containing natural extracts and fluoride prevent enamel erosion *in vitro*

**DOI:** 10.1590/1678-7757-2023-0108

**Published:** 2023-07-24

**Authors:** Tommy BAUMANN, Samira Helena NIEMEYER, Adrian LUSSI, Taís SCARAMUCCI, Thiago Saads CARVALHO

**Affiliations:** 1 University of Bern Department of Restorative, Preventive and Pediatric Dentistry School of Dental Medicine Bern Switzerland University of Bern, Department of Restorative, Preventive and Pediatric Dentistry, School of Dental Medicine, Bern, Switzerland.; 2 Center for Dental Medicine University of Freiburg Faculty of Medicine Freiburg Germany Department of Operative Dentistry and Periodontology, Center for Dental Medicine, Medical Center, University of Freiburg, Faculty of Medicine, University of Freiburg, Freiburg, Germany.; 3 Universidade de São Paulo Faculdade de Odontologia Departmento de Odontologia Restauradora São Paulo SP Brasil Universidade de São Paulo, Faculdade de Odontologia, Departmento de Odontologia Restauradora, São Paulo, SP, Brasil.

**Keywords:** Dental pellicle, Dental erosion, Enamel, Polyphenols, Mouth rinse

## Abstract

Polyphenols interact with salivary proteins and thus can improve the pellicle’s erosion protective properties. This effect could be exploited to create rinsing solutions with polyphenols as active ingredients for erosion prevention. Different from the current gold standard for erosion protective rinsing solutions, these rinses would not rely on stannous ions. This would offer alternatives for patients with concerns regarding the composition of rinsing solutions and preferring bio-products.

**Objective:**

To develop an erosion-preventive rinsing solution containing natural polyphenol-rich extracts.

**Methodology:**

Solutions were prepared with polyphenols from either grapeseed extract or cranberry extract, 500 ppm fluoride added, and additionally flavors and sweeteners. Controls were deionized water, 500 ppm fluoride solution, and the gold standard rinse in the field (Sn^2+^/F-). In total, 135 enamel specimens (n=15/group) were subjected to five cycles of salivary pellicle formation (30 min, 37°C), modification with the solutions (2 min, 25°C), further salivary pellicle formation (60 min, 37°C), and erosive challenge (1 min, 1% citric acid, pH 3.6). Relative surface microhardness (rSMH), surface reflection intensity (rSRI), and amount of calcium release (CaR) were investigated. Data were analyzed with Kruskal-Wallis and Wilcoxon rank sum tests (α=0.05).

**Results:**

The polyphenol solutions containing fluoride, as well as additional flavors, protected enamel better than fluoride alone, and similar to the Sn^2+^/F- solution, when investigating both rSMH and CaR. When measuring rSRI, Sn^2+^/F- showed the best protection, while the polyphenol solutions were similar to fluoride.

**Conclusion:**

For two of the three assessed parameters (rSMH and CaR), both developed polyphenol-rich rinsing solutions were able to protect enamel from erosion, improving/potentializing the effect of fluoride and matching the protection offered by the current gold standard rinsing solution.

## Introduction

The prevalence of erosive tooth wear (ETW) has greatly increased over the last decades. Although the increase has partially stabilized, the overall prevalence remains high.^1^ The main cause of ETW is the direct contact of the tooth surfaces with demineralizing substances. The substances can be of intrinsic or extrinsic origin,^2,3^ in which especially the increased consumption of acidic beverages has been linked to an increase in the prevalence.^4^ Since ETW is an irreversible process, which may require restorations in severe cases, effective preventive measures are needed.

The salivary pellicle, formed almost instantaneously on tooth surfaces upon contact with saliva,^5^ provides some protection from erosion.^6^ It is mainly made up of salivary proteins; however, it also contains lipids and other macromolecules from saliva, as well as bacterial components.^5^ The components of the pellicle provide a target for modifications, which can lead to improved erosion protection. Recently, several substances have been tested and shown to affect the protective properties of the pellicle in a positive way.^7-11^ While many of those modifications add new components to the pellicle, polyphenols may interact with and crosslink the existing pellicle proteins, leading to increased binding of salivary proteins to the pellicle.^12^ Thus, the pellicle gets thicker and denser,^5^ which increases its resistance to acidic attacks and limits diffusion across it. We recently showed an erosion protection by treating pellicles with natural extracts and teas, which contain large amounts of polyphenols.^11^

Polyphenols contain multiple phenol units and are divided into several subclasses. Many plants produce them as secondary metabolites involved in defense against pathogens and predation, protection from UV radiation, or attraction of pollinators. In the oral environment, polyphenols influence many sensory aspects of foods and may contribute to flavor, color, odor, bitterness, or astringency. Furthermore, they have many positive effects on health and protect against several chronic diseases like development of cancers, cardiovascular diseases, diabetes, osteoporosis, and neurodegenerative diseases.^13^

The use of polyphenol rich natural extracts has shown promising results to prevent erosion.^11^ Since polyphenols are natural products and commonly found in many foods and drinks, the acceptance for their use as preventive/therapeutic agents is likely to be high, offering a great potential for the development of erosion preventive products. Therefore, this *in vitro* study aimed to develop a rinsing solution containing such extracts and to compare its effect with the current gold standard for erosion preventive rinsing solutions.

## Methodology

### Ethics

This study was in compliance with approved guidelines and regulations of the local ethics committee (Kantonale Ethikkommission: KEK). The teeth and saliva used had been pooled and, thus, were categorized as “irreversibly anonymized” by the ethics committee. Therefore, no specific approval from the committee was necessary.

### Teeth/specimen preparation

From a pool of extracted human molars stored in 2% chloramine T trihydrate solution, 135 enamel specimens were prepared. The specimens were prepared as previously described.^11^ In brief, teeth were embedded in acrylic resin and were serially ground flat and polished with decreasing grain size, with a final polish with a grain size of 1 µm just before the start of the experimental procedure. This resulted in standardized planar parallel specimens, with the outermost 200 µm of enamel removed. Natural variations of the surface and the fluoride content that mainly occur within the outermost surface layer of enamel were thereby minimized.

### Saliva collection

Healthy donors from both sexes, from 20 to 30 years old, donated saliva. They refrained from eating or drinking for 2 h before saliva collection, which was performed in the mornings. To stimulate salivary flow the donors chewed on paraffin wax for 10 min and the stimulated whole saliva was collected in chilled vials. The saliva was pooled and centrifuged for 20 min at 4°C (4,000 g). The supernatant was divided into small aliquots and stored at -80°C until use.

### Experimental groups

**Figure 1:**
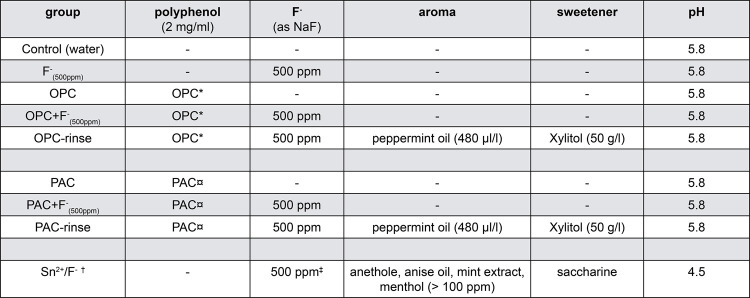
Experimental groups and their compositions. * Fairvital B.V., Germany. Content of OPC in extract: 95%; ¤ BioProphyl® GmbH (Urocyan), Germany. Content of PAC in extract: 6%; † elmex® erosion protection dental rinse; ‡ 375 ppm as NaF and 125 ppm as AmF

In total, nine experimental solutions were used. The rinsing solutions were prepared with commercially available extracts of grape seeds (OPC) and of cranberries (PAC), NaF, peppermint oil, and xylitol. Figure 1 shows the composition of the groups. All groups were prepared with deionized water. To prepare the rinses, the contents (powder) of the extract capsules were dissolved in deionized water, mixed for 30 min at room temperature, and filtered. Solutions containing extracts were prepared to contain a final concentration of 2 mg/ml of polyphenols, according to the information provided by the manufacturers of the extracts. All solutions were prepared daily and the pH of all solutions was adjusted to 5.8 with HCl or NaOH, except for the commercial Sn^2^/F^-^ solution, which was left unchanged at pH 4.5.

### Experimental design / procedure

Using the software G*Power 3.1.9.4, an effect size of 3.569 was estimated with the results from the previous study with OPC.^11^ This effect size was used for a sample size calculation with a desired power of 0.8, resulting in a sample size of n=3 per group. Expecting lower effect sizes due to adaptations to the protocol, a sample size of n=15 per group was selected. The 135 specimens were randomly allocated in the nine groups and underwent an initial assessment of the surface microhardness and reflection intensity. Then the specimens were individually subjected to five cycles consisting of: salivary pellicle formation (30 min, 37°C, no agitation), followed by treatment with the experimental rinsing solutions (10 ml, 2 min, 25°C, 70 rpm, travel path 50 mm), subsequent salivary pellicle formation (60 min, 37°C, no agitation), and an erosive challenge (10 ml, 1 min, 1% citric acid, pH 3.6, 70 rpm, travel path 50 mm). After each of these procedures, the specimens were washed with deionized water and dried with oil-free air. Between the cycles, the specimens were stored in a humid chamber. The citric acid used for erosion was stored at 4°C for subsequent calcium analyses. After each erosive challenge, the surface microhardness was measured again. Moreover, the surface reflection intensity was measured again after the final cycle and removal of the pellicle remnants.

### Surface microhardness (SMH)

Surface microhardness (SMH) was measured using a microhardness tester equipped with a Knoop diamond, with a load of 10 g for a dwell time of 10 s (UHL VMHT Microhardness Tester, UHL technische Mikroskopie GmbH & Co. KG, Asslar, Germany). SMH was measured at baseline (SMH_initial_) and after each erosive challenge (SMH_i_). Six indentations at 25 μm distance from each other were made for each measurement. The lengths of the long axes of the indentations were measured and used to calculate the hardness number, with the average of the six indentations being defined as the SMH at that time-point. The relative SMH (rSMH) at each time-point was calculated using the equation:


$$rSMH=\left(\frac{SMH_{i}}{SMH_{\text{initial }}}\right)\times 100.$$

### Surface reflection intensity (SRI)

The surface reflection intensity (SRI) was measured with a custom-built reflectometer.^14^ The maximum value of the SRI was registered with a specific software and it was measured at baseline (SRI_initial_) and after the final experimental cycle (SRI_final_). Before the final SRI measurement, the specimens were immersed in 3% NaOCl (5 min, 25°C, 70 rpm, travel path 50 mm) to remove remnants of the salivary pellicle. The relative SRI (rSRI) was then calculated according to the equation:


$$rSRI=\left(\frac{SRI_{\text{final }}}{SRI_{\text{initial }}}\right)\times 100.$$

### Calcium release (CaR)

The calcium concentration in the citric acid after the erosive challenge was analyzed using an atomic absorption spectrometer (AAnalyst 400, Perkin Elmer Analytical Instruments, Waltham, MA, USA). Lanthanum nitrate (0.5%, lanthanum nitrate hexahydrate: La[NO_3_]_3_^.^6H_2_O) was added to the citric acid to eliminate the interference of other ions. The concentration was used to determine the amount of calcium released (CaR) by each specimen. CaR was normalized to the surface area of the specimens, which was determined by taking a picture of the surface using a microscope (Leica, M420, equipped with camera DFC495) with 16 × magnification, and then tracing the contour of the exposed surface with the software program IM500.

### Statistics

The software R 3.5.3 was used for statistical analyses. All significance levels were set at α=0.05. Data of the different parameters assessed were analyzed separately. Shapiro-Wilk tests were performed to analyze whether the data were normally distributed. Since this was rejected for some groups, subsequently non-parametric tests were performed. Furthermore, Kruskal-Wallis tests were performed to analyze whether there were differences between groups. If a significant result was found, post-hoc pairwise comparisons were performed by Wilcoxon rank sum tests with Benjamini-Hochberg corrections for multiple testing.

## Results

### Hardness

Initially, absolute Knoop hardness values of all specimens ranged from 342.0 to 438.7, with values from 320 to 500 considered normal for polished enamel at the given measurement settings. The average Knoop hardness for each of the groups (median (IQR)) ranged from 395.0 (388.7-405.1) to 419.0 (406.3-423.3), without significant differences between the groups.

**Figure 2:**
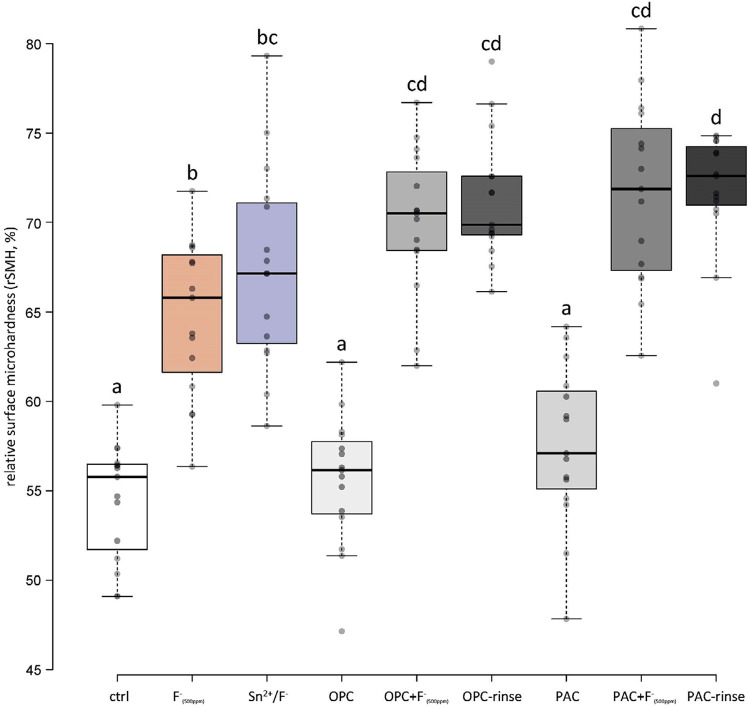
Relative surface microhardness (%) after all cycles. Different letters indicate significant differences (p<0.05)

Hardness decreased during the experiments and Figure 2 shows the final relative surface hardness (rSMH, normalized to the initial values). The extract groups alone (OPC and PAC) showed no differences to the control group, while fluoride (F^-^_(500ppm)_) and Sn^2^/F^-^ led to a significant protection. Although the extracts alone showed no effect, their combination with fluoride (OPC+F^-^_(500ppm)_ and PAC+F^-^_(500ppm)_) led to a significant protection and even performed significantly better than fluoride alone. Further addition of peppermint oil and xylitol (OPC-rinse and PAC-rinse) did not influence the effectiveness of the treatment. However, regarding PAC it even seemed to improve it slightly since this group protected significantly better than the Sn^2^/F^-^ group.

### Reflection

The initial values of SRI of the polished specimens ranged from 27.1 to 43.0. The average initial SRI of the groups (median (IQR)) ranged from 34.6 (33.4-39.0) to 39.5 (36.0-40.3), without significant differences between the groups.

**Figure 3:**
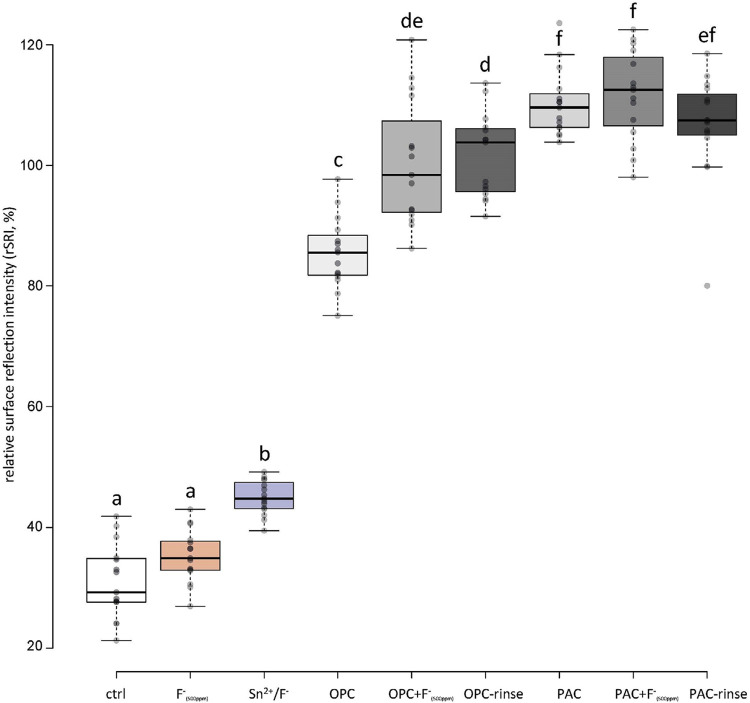
Relative surface reflection intensity (%) after all cycles, before pellicle removal. Different letters indicate significant differences (p<0.05)

**Figure 4:**
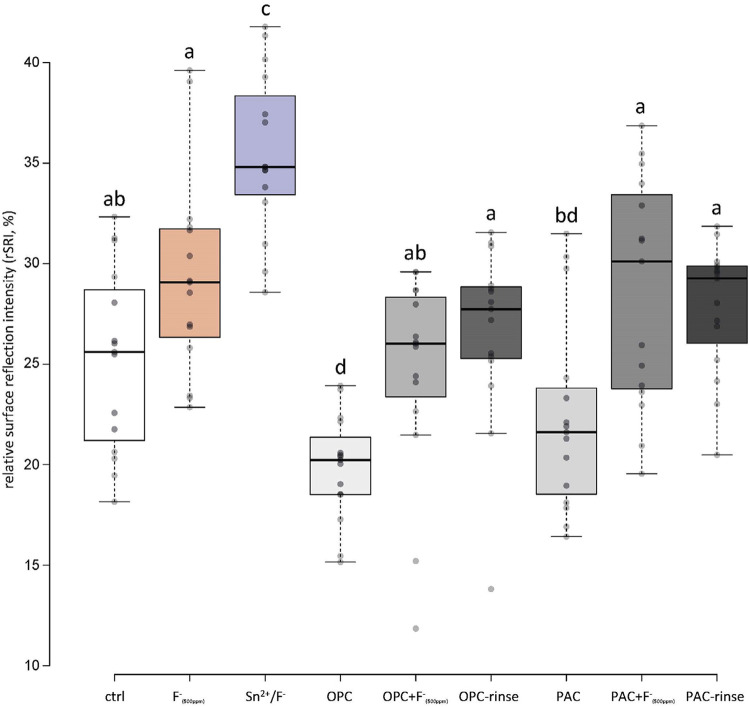
Relative surface reflection intensity (%) after all cycles, after pellicle removal. Different letters indicate significant differences (p<0.05)

As an indirect measure of surface roughness, the SRI usually decreases during erosion as the roughness of the (polished) surface increases. Figures 3 and 4 show the final relative SRIs (rSRI, normalized to the initial values). Moreover, SRI was measured first with the pellicle still present (Figure 3) and after chemically removing the pellicle (Figure 4). With the presence of pellicle, unusually high values were observed for all groups containing the extracts, while the values of the other groups decreased to different degrees. The Sn^2^/F^-^ group showed higher values than the control and F^-^_(500ppm)_ groups, without differences between the latter two, but still significantly lower values than the extract-containing groups (Figure 3). After removal of the pellicle, this changed considerably. The Sn^2^/F^-^ group showed significantly higher rSRI than all the other groups and was the only one significantly better than the control group. The groups containing extract, on the other hand, did not show significantly higher rSRI values than the control or F^-^_(500ppm)_ groups after pellicle removal (Figure 4).

### Calcium

**Figure 5:**
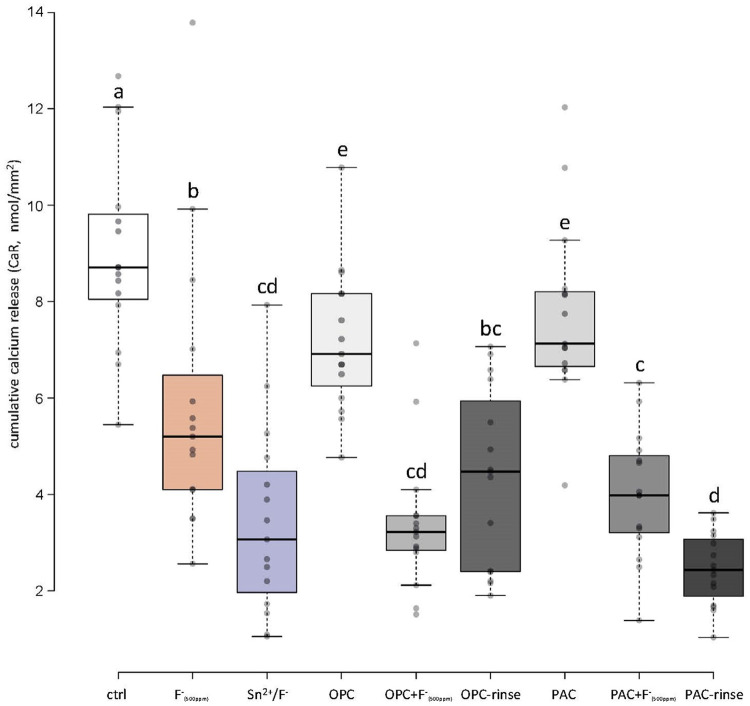
Total amount of calcium released to the citric acid. Different letters indicate significant differences (p<0.05)

Figure 5 shows the cumulative amount of calcium released from the surfaces of the specimens over all cycles of the experiment. The control group released significantly more calcium than all the other groups. This is followed by the extract-only groups (OPC and PAC), which released significantly more calcium than the remaining groups. The Fluoride group (F^-^_(500ppm)_) released significantly less calcium than the control and extract-only groups; however, it released more than the Sn^2^/F^-^ group and the extracts-combined-with-fluoride groups (except for the OPC-rinse group). Between those groups and the Sn^2^/F^-^ group, only little differences were found. Moreover, the PAC-rinse group was the only one with less calcium release than some of the extracts-combined-with-fluoride groups, but not different from Sn^2^/F^-^.

## Discussion

This study was inspired by the results of a previous study, which investigated the effect of different extracts on enamel erosion.^11^ We included two incubations with saliva in each cycle, before and after treatment with the experimental solutions, as the hypothesized mode of action is a binding of the polyphenols to the proteins of the pellicle, followed by attraction of further proteins from saliva. This is supported by a recent study showing that a periodic treatment with polyphenols leads to thicker and denser pellicles.^15^ However, different adjustments to the protocol of the previous study were necessary since we aimed to create a rinsing solution. To test whether the extracts would still be effective after those adjustments, different pre-tests were carried out (supplemental material). The pre-tests showed that the erosion inhibition decreased with decreasing treatment time from 30 min to 30 s, while decreasing the concentration of the extract from 4 to 0.25 mg/ml did not seem to have a large effect at the original treatment time of 30 min (supplemental materials). Consequently, we chose an incubation time of 2 min for the present protocol, as this treatment time still led to an erosion protection and is also a commonly used incubation time for rinsing products in *in vitro* studies.^16-18^ Regarding concentration, we chose 2 mg/ml, which represents an increase compared to the original study. Although the pre-test had showed that lower concentrations are also effective, we still chose to raise it because the treatment time was decreased to 2 min and it would still not present any concerns regarding health and safety, since much higher amounts of polyphenols are consumed in food and as food supplements.^19^ We chose the pH of 5.8 since this is the native pH of the dissolved OPC, which has previously shown a good erosion-inhibiting effect^11^ and should not cause any relevant erosion itself.

We prepared two rinsing solutions for this study. One was based on grape seed extract, containing mainly oligomeric proanthocyanidins (OPC). The other was based on cranberry extract, containing mainly general proanthocyanidins (PAC). The cranberry extract did not show good results in a previous study.^11^ In that study, the native pH was left unchanged at around 3.2, likely advancing the erosion process. Since cranberry extracts have shown promising results in other areas^20-22^ and as in proanthocyanidins it contains mainly similar polyphenols as grape seed extracts, we hypothesized that if we adjusted the pH of the cranberry extract to 5.8, the same as the pH of the grape seed extract, we could have comparable protective results compared to grape seed extract. We verified this by a pre-test, which revealed that the cranberry extract might even protect slightly better than the grape seed extract (supplemental materials). The erosion inhibiting action of the polyphenols is likely based on their interaction with proteins of the pellicle, especially from the basal layer of the pellicle.^23^ This could, in turn, attract more proteins to the pellicle, leading to changes in its structure and protective properties.^24,25^

Although fluoride alone shows some effect, its success in erosion inhibition is limited.^26^ However, fluoride is important for caries prevention. The main sought-after function of the rinses created in this study was erosion prevention; however, we included fluoride in the rinses for its possible additional erosion inhibiting effect and considered the essential prevention of caries. The results show that fluoride alone provides some erosion protection with the *in vitro* protocol that we used, as it led to a higher rSMH and less calcium release than the control. Only the rSRI did not differ from the control group, regardless if measured with the pellicle still present or after pellicle removal. Combining the extracts with fluoride led to even significantly better protection, with higher rSMH and less calcium release than fluoride alone, suggesting a synergistic effect. This was also demonstrated in a recent study inspired by this finding.^27^

Peppermint oil and xylitol were added to the rinses to improve the taste. They were not thought to influence erosion inhibition, which was generally confirmed by our results. Neither the rSMH nor rSRI showed any differences between the groups containing extracts and fluoride (OPC+F^-^_(500ppm)_ and PAC+F^-^_(500ppm)_) and the groups containing additionally oil and xylitol (OPC-rinse and PAC-rinse; Figures 1 and 3). Only the calcium release showed an additional reduction for the PAC with those additives. Lipids are also part of the pellicle,^28,29^ and its lipid profile can be modified.^30^ Since the lipid content of our rinsing solutions is low, a modification of the lipid profile was not expected but we cannot exclude it. Polyphenols also interact with lipids.^31^ Therefore, PAC might also influence the lipid profile of the pellicle, which might have a positive effect on erosion protection; however, we did not further investigated this.

The two rinsing solutions prepared were compared to the current gold standard of erosion protective rinsing solutions, a commercially available Sn^2^/F^-^ containing solution.^17^ The results show that, under the rather mild conditions used, the OPC-rinse and the PAC-rinse performed comparable to the Sn^2^/F^-^ solution. Regarding rSMH, all three rinses showed a significant erosion inhibition compared to the control, with little differences between themselves. The OPC-rinse and PAC-rinse performed better than the fluoride (F^-^_(500ppm)_) group, while the PAC-rinse even exhibited a significantly higher final rSMH than the Sn^2^/F^-^ group (Figure 2). The calcium release results were similar. All three rinses significantly reduced the amount of calcium released compared to the control, without differences between themselves (Figure 5). Only in rSRI, the Sn^2^/F^-^ group seemed to perform better, as after pellicle removal it exhibited higher values than the experimental rinses (Figure 4). The rSRI is an indirect measure of the surface roughness, with smoother surfaces leading to higher reflection values. After a certain degree of erosion the surface roughness, as measured via the rSRI, does not change a lot anymore.^14^ On the other hand, calcium release from near-surface layers might still vary considerably,^32^ as well as the resulting change in hardness. If the surface is not directly modified by a treatment, then there are no differences observable in the rSRI at this stage. This is the case for the polyphenol groups since the polyphenols do not bind to and act directly on the surface, but instead they interact with the proteins of the pellicle,^11,12^ leading to thicker and denser pellicles.^15^ The Sn^2^/F^-^ group, on the other hand, contains stannous ions as the main active ingredient. Those ions are incorporated into the top layer of eroded enamel,^33^ which could lead to differences in the smoothness and, thus, the reflection of the surface. The better performance of the Sn^2^/F^-^ group observed in rSRI might therefore be due to the combination of differences in the mode of action and the measurement technique rather than a real increased protection compared to the other rinses. A disadvantage that increases the difficulty in interpreting the results from the SRI measurements is that the pellicle removal with the protocol used might be incomplete. A validated protocol for complete pellicle removal requires 30 min of incubation in NaOCl, together with sonication.^34^ However, sonication damages the eroded enamel,^35^ thus we chose not to follow this protocol. Although pellicle remnants might be left on the surface, we clearly observed differences between before (Figure 3) and after (Figure 4) pellicle removal.

Polyphenols are sensitive to light,^36^ which could be a problem for the stability and the shelf-life of a rinsing solution. Therefore, we carried out an additional pre-test for the shelf-life of OPC+F^-^_(500ppm)_ and the OPC-rinse, storing them for five months at room temperature in the dark. After this storage period the erosion preventive effect of the solutions was still maintained (supplemental materials).

Up to now, we observed promising results for our experimental solutions. However, they were so far tested only under *in vitro* conditions, so a next step will be to test these rinsing solutions in a clinical setting. This is especially important because the mechanism of action of polyphenols is based on interactions with proteins from the salivary pellicle and there are differences between *in vitro* and *in situ* or *in vivo* pellicles.^37^ Considering the high affinity of the OPC and PAC to the salivary proteins, we believe that the polyphenol rinses will also be effective in these situations. A further step is to test the solutions in a harsher erosion model to verify how it performs under more severe challenges. Furthermore, a protocol including abrasion should be tested to investigate how the rinses perform under ETW conditions rather than solely demineralizing conditions. A concern that polyphenols share with stannous-based products is that they might cause tooth staining.^38^ However, it was recently demonstrated that polyphenol-based staining is reversible.^39^ To find alternatives to stannous-based products, which are so far the gold standard despite some disadvantages, other efforts to create erosion protective rinses are based on polymers.^40^ Although some of these polymers are also essentially natural products, an advantage of the present OPC and PAC rinses is that they are easily recognized as based on natural plant-based products. This might improve acceptance and compliance of some patients to use these rinses.

## Conclusion

Considering our results, we created a natural erosion preventive rinsing solution containing OPC or PAC. Under the *in vitro* conditions tested in this study, these rinsing solutions perform as well as or even better than the current gold standard for prevention of initial erosive demineralization. This offers alternatives in erosion prevention, as the mechanism of action of the polyphenol-based products is different, exploiting the natural interaction of polyphenols with salivary proteins, thus modifying and strengthening the pellicle.
